# The Association Analysis of Vascular Endothelial Growth Factor -2549 Insertion/ Deletion Variant and Endometriosis Risk

**DOI:** 10.22088/IJMCM.BUMS.8.2.63

**Published:** 2019-05-28

**Authors:** Negar Sarhangi, Shahrzad Mohseni, Soheila Aminimoghaddam, Batool Hossein Rashidi, Fedyeh Haghollahi, Mostafa Qorbani, Mahsa Mohammad Amoli, Maryam Shahrabi-Farahani

**Affiliations:** 1 *Personalized Medicine Research Center, Endocrinology and Metabolism Clinical Sciences Institute, Tehran University of Medical Sciences, Tehran, Iran.*; 2 *Metabolic Disorders Research Center, Endocrinology and Metabolism Molecular-Cellular Sciences Institute, Tehran University of Medical Sciences, Tehran, Iran.*; 3 *Endocrinology and Metabolism Research Center, Endocrinology and Metabolism Clinical Sciences Institute, Tehran University of Medical Sciences, Tehran, Iran.*; 4 *Department of Obstetrics and Gynecology, Iran University of Medical Sciences, Tehran, Iran.*; 5 *Vali-e-Asr Reproductive Health Research Center, Tehran University of Medical Sciences, Tehran, Iran.*; 6 *Department of Community Medicine, School of Medicine, Alborz University of Medical Sciences, Karaj, Iran. *

**Keywords:** Endometriosis, vascular endothelial growth factor, VEGF, -2549 I/D, polymorphism

## Abstract

Endometriosis is a debilitating disorder, defined as the presence of endometrial gland and stroma outside of the uterus. It may affect angiogenesis and *vascular endothelial growth factor (VEGF)* is one of the angiogenic factors that plays an important role in both physiological and pathological angiogenesis. The present study aimed to evaluate the association of *VEGF* -2549 insertion/deletion (I/D) polymorphism with endometriosis. This case-control study enrolled 244 (100 cases and 144 controls) women who were admitted for laparoscopy or laparotomy for gynecological procedures. Genomic DNA was separated from peripheral blood leukocytes and polymerase chain reaction (PCR) amplification was performed for genotyping of the *VEGF* gene Insertion/Deletion (I/D) polymorphism. The frequency of the II, ID, and DD genotype was 14%, 52% and 34% in patients versus 18.8%, 47.8% and 34% in controls. The results did not provide any evidence supporting the endometriosis risk related to the *VEGF* polymorphism in a group of Iranian women population.

Endometriosis is a debilitating disorder, with a multifactorial pathogenesis, in which the endometrial gland and stroma that normally lines the inside of the uterine cavity, grows outside of the uterus, principally on the pelvic peritoneum, ovaries, and rectovaginal septum (1, 2). Approximately 6-10% (3) of women in their reproductive period, and 20-40 % (4) of women who may not be able to have children are affected by endometriosis. It is manifested by pelvic pain, dyspareunia, painful bowel movement, and painful urination. Other signs of disease are bleeding between two menstruation cycles and infertility (5). A heritable tendency of the disease has been established, in which, in the first-degree of affected women the risk of developing endometriosis is 7 times higher than the relatives of unaffected women (6). Angiogenesis and inflammatory response are thought to be playing a key role in the development of endometriosis. The *vascular endothelial growth factor* (*VEGF*), also known as vascular permeability factor, is one of the most important regulators of angiogenesis (7). The human *VEGF* gene is located on chromosome 6p21.3 (8) and consists of 8 exons that is the result of different alternative splicing from a family of proteins (9). *VEGF-A*, the most predominant *VEGF* isoform, has q highly polymorphic promoter and 5'-untranslated region (5'-UTR) (8). Several functional polymorphisms in these regions as well as the 3'-UTR of the *VEGF* gene have been described to be associated with variation in VEGF protein production and have been related to several disorders in which angiogenesis is important in the progression of the disease (10). Some studies indicated the role of Insertion/ Deletion (I/D) polymorphism at -2549 position of the promoter region in *VEGF* gene which is associated with several disorders such as breast cancer (8), end stage renal disease (ESRD) (11), peripheral arterial disease (PAD) in type 2 diabetes mellitus (T2DM) (12), diabetic nephropathy (DN) (13, 14) and retinopathy (DR) (15, 16), asthma (17), prostate cancer (18), and Behcet's disease (BD) (19) in which angiogenesis may be implicated. So far, the association of I/D polymorphism of the 18 bp fragment, at -2549 position of the promoter region in *VEGF* gene to endometriosis has not been investigated to date. In this study, we sought to investigate the association between -2549 I/D *VEGF* polymorphism and susceptibility to endometriosis in a group of Iranian women population for the first time.

## Material and Methods

**Subjects**

Two hundred forty-four women who were admitted for laparoscopy or laparotomy for gynecological procedures in two university hospitals of Tehran, Iran from April 2013 through September 2015 were considered eligible. The study design was approved by the Endocrinology and Metabolism Research Institute, affiliated to Tehran University of Medical Sciences (TUMS), and written informed consent was obtained from all participants.

Demographic data, gynecological and obstetrical history such as parity, menstrual cycle, and dysmenorrhea were obtained by interviews during preoperative appointments. Subjects were considered as cases (n=100) if they had visible ectopic implants, and a histologically confirmed diagnosis of endometriosis after their operative findings, according to the presence of endometrial glands or stroma in the lesions. The control group (n=144) consisted of women undergoing laparoscopy or laparotomy for tubal ligation or treatment of benign diseases such as ovarian cysts, myoma, hydrosalpinx, etc., and who had no macroscopic signs of endometriosis at surgery. The patients with no history of malignant disease and who had not received hormonal treatment for at least 6 months from the date of sampling were included in the study.

**Sample collection, DNA extraction, and** ***VEGF*** **polymorphism evaluation**

For DNA extraction, 3 ml of peripheral blood (in EDTA containing vial) was obtained from each study subject at the time of enrollment and stored at -20°C.

Genomic DNA was separated from peripheral blood leukocytes using phenol protocol. In this study, I/D polymorphism of 18 bp fragment at -2549 position of the promoter region in *VEGF* was selected to investigate. The polymerase chain reaction (PCR) assay was used directly for the detection of this polymorphism.

The I/D polymorphism was analyzed using the common set of forward 5'- GCTGAGG ATGGGGCTGACTAGGTA-3' and reverse: 5'-GTTTCTGACCTGGCTATTTCCAGG-3' primers as indicated by Buraczynska et al. (15). PCR amplification was accomplished in 25 µl final volume containing 1 µl template DNA, 10 µl Red Master Mix (Amplicon, UK), 0.7µl each primer and 12 µl H_2_O. The allele/detection process was performed on a Biorad MJ mini (Singapore) to determine the allelic discernment. Thermal cycling was initiated with the first denaturation step of 6 min at 95°V, followed by 35 cycles of denaturation at 95°C for 30 s and annealing at 72°C for 10 min. The amplification products were segregated by electrophoresis on 2.5% agarose gel stained with SYBR green. For the *VEGF* I/D polymorphism, two bands were observed, a band of 229 bp represents an 18 bp insertion (I allele), and the band of 211 bp represents an 18 bp deletion (D allele). About 5% of the accidentally selected samples were confirmed by sequencing.

**Statistical analysis**

Data were analyzed using Statistical Package for Social Sciences software, version 16.0 (SPSS, Chicago, IL). Continuous variables were expressed as mean ± standard deviation (SD), and categorical variables were expressed as number and percentage. Student’s t-test was used for comparing quantitative variables and the Chi-squared test was used to analyze categorical data.

The difference of genotype and allele frequencies between endometriosis patients and controls was assessed by regression test, and an odds ratio (OR) with 95% confidence interval (CI) was used as a measure of the strength of association between genotypes and allele frequencies. Probability values less than or equal to 0.05 were considered statistically significant.

## Results

Table 1 shows the baseline characteristics of the study population. Both groups were age matched (P =0.17). On the contrary body mass index (BMI), dysmenorrhea, gravidity and infertility were significantly different between the two groups (P<0.01). No significant differences were found with regard to bleeding volume and irregular menses between two groups.

**Table 1:** Baseline characteristics data of study population

**Variables**	**Cases (N=100)**	**Controls (N=144)**	P-value*
**Age (years)**	35.39±7.56	37.11±10.9	0.17
**BMI**	25.47±4.66	27.96±4.21	<0.01
**Gravity** Yes	47(47)	130(90.3)	<0.01
**Infertil**ity	51(51)	9(6.2)	<0.01
**Dysmenorrhea**	68(69.4)	56(40.3)	<0.01
**Bleeding volume**			
Low Moderate High	11(11.2) 58 (55) 29(29.6)	11(7.9) 77(52.9) 52(37.1)	0.39
**Irregular menses** (<21 or > 35 days Interval)	28(28)	41(28.5)	0.49


**Table 2:** Genotype and allele distributions of -2549 I/D between two groups, and endometriosis risk of VEGF polymorphism

**Genotypes**	** Cases** **N (%)**		** Controls** **N (%)**		**OR (95%CI)**	**P-value**
**II**	14	14	27	18.8	1 (Reference)	
**ID**	52	52	68	47.8		
**Crude**					1.47 (0.7-3.09)	0.3
**Adjusted					1.32 (0.6-2.88)	0.47
**DD**	34	34	49	34		
**Crude**					1.33 (0.61-2.91)	0.46
**Adjusted					1.16 (0.51-2.66)	0.71
**Alleles**						
**I**	66	66	95	66	1 (Reference)	
**D**	34	34	49	34		
**Crude**					0.99 (0.58-1.71)	0.99
**Adjusted					0.94 (0.52-1.69)	0.85


The proportion of patients suffering from endometriosis symptoms such as dysmenorrhea was significantly higher in cases in comparison with the control group (P <0.01).

A total of 244 (100 cases and 144 controls) of the enrolled subjects were successfully genotyped for *VEGF* polymorphism. The genotype and allele frequency of the -2549 I/D *VEGF* gene polymorphism among controls and cases are shown in Table 2.

Table 2 shows the endometriosis risk related to *VEGF* polymorphism. No significant differences were found in allele and genotype distribution of the *VEGF* -2549 I/D polymorphism between cases and controls even after adjustment for age and body mass index (BMI).

## Discussion

Our results do not provide any evidence supporting an association between this *VEGF* polymorphism and endometriosis susceptibility. Previous studies indicated that the DD genotype of -2549 I/D polymorphism has been associated with diabetic nephropathy (13, 14), and peripheral arterial disease in T2DM (12).

Yang et al. (13), showed that the D allele and D/D genotype of the *VEGF* may be associated with susceptibility to diabetic nephropathy. The pattern including the 18 bp deletion (D allele) had a 1.95-fold increase in the transcriptional activity compared with the other allele containing the inserted construct (I allele).

Among the North Indian population, DD genotype at -2549 region of the *VEGF* gene has been associated with 1.58-fold increased risk of end-stage renal disease (ESRD) (11).

In a study by Fouad et al. (20), no significant association was perceived between I/D polymorphism in the promoter region of the *VEGF* gene and retinopathy in Egyptian T2DM patients. However, DD genotype of -2549 I/D polymorphism had>2- fold and D allele had >1.5 –fold raised risk in patients with diabetic retinopathy than controls.

Buraczynska et al. (15) identified the association between the DD genotype and the D allele with retinopathy but not nephropathy in type 2 diabetic patients. Some reports showed that, this polymorphism at -2549 position of the *VEGF* gene is in complete linkage with -2578 A/C polymorphism (21). It has been showed that individuals with the -2578A allele have an 18 bp insertion, but those with - 2578 C allele have not (13).

The results of the study by Kapahi et al. (8) in North India suggested that -2549 I/D polymorphism was associated with susceptibility to breast cancer. On the contrary, some studies did not perceive any association between ID and DD genotypes with recurrent miscarriages in the North Indian population (22), systemic sclerosis in the European Caucasians (23), and hepatocellular carcinoma (HCC) in the Chinese Han population (24). On the other hand, this genotype showed >2- fold risk in patients with prostate cancer (18). Salvarani et al. (19) showed that the I allele of -2549 I/D polymorphism was associated with Behcet's disease (BD) in Italian patients.

In conclusion, we did not find a statistically significant association in *VEGF* alleles frequencies between the endometriosis and non-endometriosis Iranian women.

Prospective studies in larger endometriotic patient populations, as well as the functional studies, are needed to elucidate the role of the *VEGF* I/D polymorphism in endometriosis disease.
